# Pyogenic Liver Abscess and Risk of Acute Pancreatitis

**DOI:** 10.2188/jea.JE20150075

**Published:** 2015-07-05

**Authors:** Hsiu-Nien Shen

**Affiliations:** 1 Department of Intensive Care Medicine, Chi Mei Medical Center, Tainan, Taiwan; 2 Department of Public Health, College of Medicine, National Cheng Kung University, Tainan, Taiwan

To the Editor:

I read with interest the recent article by Lai et al on risk of acute pancreatitis after discharge from hospitalization for pyogenic liver abscess (PLA).^1^ In this cohort study, the authors found that PLA was associated with an increased risk of acute pancreatitis compared to non-PLA (adjusted hazard ratio 3.00; 95% confidence interval, 2.62–3.43).^1^ However, the estimate may not be valid because of a violated proportionality assumption, which was acknowledged by the authors.^1^ To deal with non-proportional hazards, it would be more appropriate to use time partitioning or other methods for time-varying covariates,^2^ instead of modeling using different follow-up periods (as shown in Table 2 of the article).^1^ Moreover, the interaction effects of PLA and comorbidity on risk of acute pancreatitis (Table 3 of the article)^1^ may differ by time-partitioned period.^2^
